# Patient-, Provider-, and Facility-Level Contributors to the Use of Cardiology Telehealth Care in the Veterans Health Administration: Retrospective Cohort Study

**DOI:** 10.2196/53298

**Published:** 2024-10-25

**Authors:** Rebecca Lauren Tisdale, Jacqueline M Ferguson, James Van Campen, Liberty Greene, Charlie M Wray, Donna M Zulman

**Affiliations:** 1 Center for Innovation to Implementation Veterans Affairs Palo Alto Health Care System Menlo Park, CA United States; 2 Division of Primary Care and Population Health Department of Medicine Stanford University School of Medicine Stanford, CA United States; 3 Veterans Affairs San Francisco San Francisco, CA United States; 4 Department of Medicine University of California, San Francisco San Francisco, CA United States

**Keywords:** telehealth, mixed models, veteran, patient, virtual cardiology, cardiology care, cohort study

## Abstract

**Background:**

Telehealth (care delivered by phone or video) comprises a substantial proportion of cardiology care delivered in the Veterans Health Administration (VHA). Little is known about how factors specific to patients, clinicians, and facilities contribute to variation in cardiology telehealth use.

**Objective:**

The aim of this study is to estimate the relative extent to which patient-, clinician-, and facility-level factors affect cardiology telehealth use in VHA.

**Methods:**

This was a retrospective, nation-wide cohort study of veterans’ use of VHA cardiology telehealth care during the first 2 years of the COVID-19 pandemic (March 11, 2020, to March 10, 2022). We constructed multilevel, multivariable, logistic regression models of patient-level cardiology telehealth use (telephone or video-based care). Models included random effects for the patient, the patient’s main cardiology provider, and the patient’s primary facility (ie, VHA medical center) for specialty care and fixed effects for patient sociodemographic and clinical characteristics.

**Results:**

Our analytic cohort comprised 223,809 veterans with 989,271 encounters among 2235 unique clinicians. The veterans’ average age was 70.2 years, and 3.4% (n=7616) were women. Of the 989,271 encounters, 4.2% (n=41,480) were video based and 34.3% (n=338,834) were phone based. Adjusted odds of telehealth use were slightly higher for women versus men (adjusted odds ratio [AOR] 1.08, 95% CI 1.05-1.10), individuals identifying as Hispanic or Latino versus not Hispanic or Latino (AOR 1.46, 95% CI 1.43-1.49), and those with medium and long drive times versus short drive time (AOR 1.11, 95% CI 1.10-1.12 and AOR 1.09, 95% CI 1.07-1.10, respectively). Further, 40.5% of the variation in a veteran’s likelihood of using cardiology telehealth care was found at the patient level, 30.8% at the clinician level, and 7% at the facility level.

**Conclusions:**

The largest share of the attributable variability in VHA cardiology telehealth use in this cohort was explained by the patient, followed closely by the clinician. Little variability was attributed to the primary facility through which the veteran received their cardiology care. These results suggest that policy solutions intended to improve equity of cardiology telehealth care use in VHA may be most impactful when directed at patients and clinicians.

## Introduction

The coronavirus (COVID-19) pandemic has been a catalyst for the expansion of telehealth (medical care delivered by phone or video), in the Veterans Health Administration (VHA) system and in health care settings worldwide [1,2]. While in-person care has largely been restored as the pandemic has subsided, telehealth continues to play a crucial role in improving access to medical care. However, as telehealth shifts from a requirement to an option [3], this role is evolving; beyond replacing an in-person encounter, telehealth can be used to address staffing gaps at a given location. For example, VHA has implemented a hub-and-spoke model across primary, mental health, and specialty care to compensate for staffing shortages, wherein clinicians at the “hub” site see patients at a “spoke” sites via telehealth [4].

For optimal and equitable use, such novel applications of telehealth depend on a thorough understanding of the drivers of telehealth use. Unsurprisingly, these drivers may differ by medical specialty and clinical conditions. Acknowledging this, professional societies have called for more research into determinants of telehealth provision for cardiovascular disease specifically [5]. Use of cardiology telehealth care varies depending on patients’ sociodemographic characteristics, such as age, rurality, socioeconomic status, and number of comorbid conditions [6,7]. At the clinician level, significant variability exists regarding comfort level with and support for telehealth adoption among cardiologists [8]. Additionally, facility-level characteristics have also been shown to influence telehealth adoption [9]. Given that all 3 levels are likely influencing telehealth use, understanding the *relative* contribution of each is useful for informing decisions about where to direct policy change for the highest impact [10]. While other studies have examined this question across a range of specialties [11], none has specifically focused on cardiology telehealth care.

In this study, we estimate the variability in cardiology telehealth care use attributable to patients, clinicians, and health care facilities. Understanding the relative contribution of factors at these levels can inform policy initiatives and interventions to promote the optimal and equitable use of telehealth in specialty settings.

## Methods

### Data

This retrospective cohort study focused on active users of VHA cardiology care. Veterans aged 18 and older were included in the cohort if they had at least 1 outpatient cardiology visit for a cardiology diagnosis (the full list of diagnoses is provided in Table S1 in Multimedia Appendix 1) in the approximately 15-month period leading up the COVID-19 pandemic’s onset (calendar year 2019 and the beginning of 2020; January 1, 2019, to March 10, 2020) and had at least 2 outpatient cardiology visits in the first 2 years of the pandemic (March 11, 2020, to March 10, 2022). The first day of the pandemic was considered to be March 11, 2020, consistent with World Health Organization directives [12].

We sourced all data from VHA’s Corporate Data Warehouse, a repository for veteran’s electronic health care records (172VA10P2: VHA Corporate Data Warehouse - VA 79 FR 4377). In addition to veterans’ dates of birth and, when applicable, dates of death, the dataset included all cardiology outpatient encounters in VHA, as defined by VHA-specific “stop codes,” where the visits were associated with an independent licensed practitioner with a unique National Provider Identifier (NPI). These 3-digit codes, available through VHA’s Managerial Cost Accounting system, characterize all VHA outpatient encounters and associated clinical work units (Table S2 in Multimedia Appendix 1). Stop codes were also used to define whether the visit was conducted in person or via phone or video, with telehealth visits defined as those taking place by phone or video. Visits for cardiac rehabilitation were not included.

We captured veteran sociodemographic and clinical characteristics in a manner consistent with existing VHA telehealth literature [1,2,6]. Age at the beginning of the pandemic was categorized into 4 groups, roughly corresponding to quartiles for the study population: <50, 50-64, 65-74, and ≥75 years. Race was categorized as American Indian or Alaska Native, Asian, Black or African American, Native Hawaiian or other Pacific Islander, unknown, or White, and ethnicity was categorized as Hispanic or Latino, not Hispanic or Latino, or unknown, both based on the most frequently recorded race or ethnicity identification in the electronic health record. Separate categories were created for missing race or ethnicity classification. Rurality, defined as highly rural (population density of fewer than 7 people per square mile), rural, or urban, was based on US Census Bureau criteria [13] and derived from VHA’s Planning Systems Support Group (PSSG) Geocoded Enrollee Files in the Corporate Data Warehouse. Drive time to secondary care, which includes cardiology care, was also sourced from PSSG files and was categorized as short (≤30 min), medium (31-60 min), or long (>60 min). We included VHA enrollment priority category as a measure of social and medical risk; this system groups veterans based on military service–connected disability, recent military service, income, and other factors [14]. As in prior work [1,6], the 8 enrollment priority categories were condensed into 4: high disability (>50% service-connected disability or VHA catastrophically disabled), low/moderate disability (10%-40% service-connected disability or military exposure), low income (annual income below area-adjusted mean), or without special VHA enrollment priority. Noncardiology care was captured as primary care visits in the year prior to the study period and categorized into tertiles thereof (0-4, 5-8, or ≥9). Use of mental health care and emergency department or urgent care visits in the year prior to the analysis period were included as binary variables. Veteran chronic conditions were calculated out of a predefined group of 47 possible *International Classification of Disease, Tenth Revision* (*ICD-10*) diagnosis groups, constructed in prior studies in VHA’s population [15-18] (Table S3 in Multimedia Appendix 1). Housing instability was based on a combination of outpatient stop codes denoting use of Veterans Affairs housing services and diagnosis codes (Table S2 in Multimedia Appendix 1). Calendar year (2020, 2021, and 2022) was also included.

While in practice patients may see multiple clinicians (including, eg, a nurse practitioner and a physician, or a trainee and an attending physician), sometimes across different facilities, for the purposes of this analysis, we assigned patients a primary cardiology clinician and both patients and clinicians a primary facility. If patients had cardiology encounters with multiple independent licensed practitioners, we defined their main cardiology provider as the provider they had seen most often, or in the case of ties, most recently. In this cohort, 25.1% (56,176/223,809) of patients saw a single cardiology provider; 27.9% (62,443/223,809/N) saw 2, and 47% (105,190/223,809) saw 3 or more cardiology providers. Likewise, for patients seen at multiple VHA medical centers (ie, facilities) for cardiology care, the patient’s home site was the site at which the majority of their cardiology encounters took place. In total, 92.6% (207,247/223,809) of patients received care from a single VHA medical center, and 99.6% (222,914/223,809) received care from 2 or fewer.

### Statistical Analysis

In addition to descriptive statistics for patient characteristics, we constructed multilevel logistic regression models of our primary outcome, a patient’s odds of receiving cardiology telehealth care (ie, care delivered by phone or video). These models included random effects for the patient, the patient’s main cardiology provider, and the patient’s home VHA facility for specialty cardiology care. Models were adjusted for the patient sociodemographic and clinical characteristics delineated above and calendar year as fixed effects. Statistical analyses were conducted in Stata 17 (StataCorp, LLC).

### Ethical Considerations

This analysis was carried out as part of the Virtual Access Quality Enhancement Research Initiative, which is designated as nonresearch quality improvement by VHA program office partners in the VHA Office of Rural Health. The institutional review board at the Stanford Research Compliance Office determined this evaluation does not meet the requirement of research or clinical investigation per Federal Regulations 45CFR 46.104 (Subsection 4) [19] and VA 38CFR 16.104 (Subsection 4) [20]. To protect the privacy and confidentiality of human subjects, study data were anonymous.

## Results

**Figure 1:**
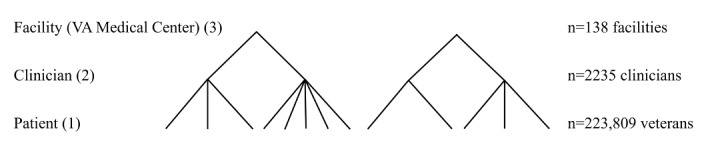
Structure of multilevel model and the number of contributors at each level (based on the model from Leyland and Groenewegen [21]). VA: Veterans Affairs.

Our analytic cohort comprised 989,271 encounters for 223,809 veteran patients, among 2235 clinicians and 138 facilities (VHA medical centers). Figure 1 [21] illustrates the structure of a basic 3-level multilevel statistical model [21] and the numbers included at each level.

Figure 1 depicts examples of how varying numbers of patients and clinicians may be grouped under a given facility (in this case, a Veterans Affairs medical center). Numbers in parentheses denote the level numbers in the model.

Overall, among the 989,271 encounters, 4.2% (n=41,480) of encounters were video based and 34.3% (n=338,834) were phone based. Of these, 385,707 (39%) were virtual (telehealth) over the 2-year period, although this dropped from 52.5% (278,053/529,401) of encounters being conducted virtually in the first year of the pandemic to 23.4% (107,654/459,870) in the second year. Of these veterans, 161,424 had at least 1 telehealth (video or phone) visit (Table 1). The majority of these individuals (n=137,004, 84.9%) used only the phone option for telehealth, as there were only 24,420 video care users.

The average age for veterans in the overall cohort and the subset of telehealth users was the same: 70.2 years (Table 1). Users of video care were a bit younger, with an average age of 68.7 years. Women comprised 3.4% (7616/223,809 and 5528/161,424, respectively) of the overall and telehealth groups. Most sociodemographic and clinical characteristics were similar between the general cohort and the subset of telehealth users, including proportions of each racial or ethnic group, rurality, drive time to care, enrollment priority, use of prior care, and numbers of chronic conditions.

Veterans and providers varied considerably in their encounters per person (Figure S1 in Multimedia Appendix 1). For patients, the mean number of encounters was 4 (SD 3), and for providers, the mean number of encounters among this patient cohort was 443 (SD 648). The mean patients per facility was 1749 (SD 1139) and the mean cardiology providers per facility was 17 (SD 14).

**Figure 2:**
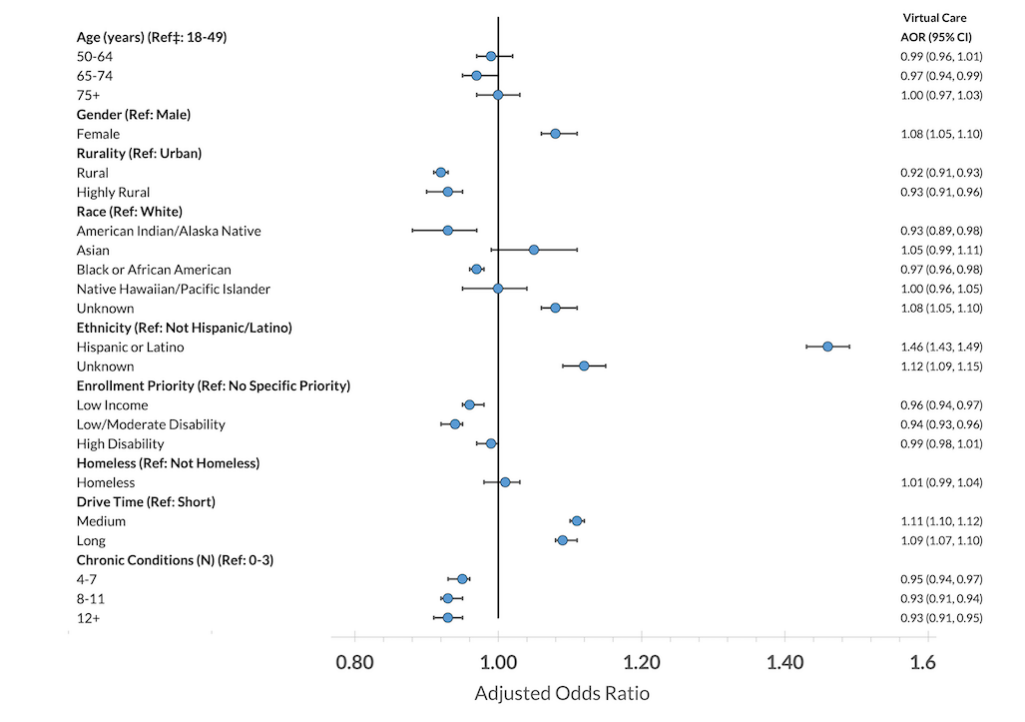
Adjusted odds of using cardiology telehealth care among established cardiology patients, selected characteristics (N=223,809). Full model results are included in Table S4 in Multimedia Appendix 1. AOR: adjusted odds ratio.

In adjusted multilevel models with random intercepts for the patient, patient’s main clinician, and patient’s home site, most differences by patient characteristics were small in magnitude if present (Figure 2; Table S4 in Multimedia Appendix 1).

**Table 1:** Characteristics of the study population (N=223,809) and telehealth users.

Characteristics			All patients^a^ (N=223,809)		Telehealth^b^ users (n=161,424)		Telehealth nonusers (n=62,385)
Age (years), mean (SD)			70.2 (9.6)		70.2 (9.6)		70.1 (9.4)
**Age group (years), n (%)**							
	18-49	6646 (3)		4838 (3)		1808 (2.9)	
	50-64	44,218 (19.8)		31,957 (19.8)		12,261 (19.7)	
	65-74	110,813 (49.5)		79,447 (49.2)		31,366 (50.3)	
	75+	62,132 (27.8)		45,182 (28)		16,950 (27.2)	
**Race, n (%)**							
	American Indian or Alaska Native	1865 (0.8)		1304 (0.8)		561 (0.9)	
	Asian	1310 (0.6)		981 (0.6)		329 (0.5)	
	Black or African American	36,180 (16.2)		26,477 (16.4)		9703 (15.6)	
	Native Hawaiian or other Pacific Islander	1937 (0.9)		1420 (0.9)		517 (0.8)	
	Unknown	9572 (4.3)		7033 (4.4)		2539 (4.1)	
	White	172,945 (77.3)		124,209 (76.9)		48,736 (78.1)	
**Ethnicity, n (%)**							
	Not Hispanic or Latino	206,846 (92.4)		148,404 (91.9)		58,442 (93.7)	
	Hispanic or Latino	10,574 (4.7)		8225 (5.1)		2349 (3.8)	
	Unknown	6389 (2.9)		4795 (3)		1594 (2.6)	
**Gender, n (%)**							
	Women	7616 (3.4)		5528 (3.4)		2088 (3.3)	
	Men	216,193 (96.6)		155,896 (96.6)		60,297 (96.7)	
**Rurality, n (%)**							
	Urban	144,217 (64.4)		105,038 (65.1)		39,179 (62.8)	
	Rural	70,350 (31.4)		49,891 (30.9)		20,459 (32.8)	
	Highly rural	9242 (4.1)		6495 (4)		2747 (4.4)	
Drive time to secondary care (min), mean (SD)			43.6 (34.0)		43.2 (33.3)		44.9 (35.6)
**Drive time to secondary care, n (%)**							
	Short	98,295 (43.9)		70,897 (43.9)		27,398 (43.9)	
	Medium	71,178 (31.8)		52,369 (32.4)		18,809 (30.1)	
	Long	52,708 (23.6)		37,025 (22.9)		15,683 (25.1)	
	Missing	1628 (0.7)		1133 (0.7)		495 (0.8)	
**Enrollment priority, n (%)**							
	No service disability	27,791 (12.4)		19,912 (12.3)		7879 (12.6)	
	Low/moderate disability	42,037 (18.8)		29,791 (18.5)		12,246 (19.6)	
	High disability	100,122 (44.7)		72,309 (44.8)		27,813 (44.6)	
	Low income	53,859 (24.1)		39,412 (24.4)		14,447 (23.2)	
**History of housing instability, n (%)**							
	No	215,367 (96.2)		155,211(96.2)		60,156 (96.4)	
	Yes	8442 (3.8)		6213 (3.9)		2229 (3.6)	
Primary care visits in year prior to analysis period, mean (SD)			7.2 (6.6)		7.3 (6.7)		6.9 (6.2)
**Primary care visits in year prior to analysis period, n (%)**							
	0-4	93,879 (41.9)		66,452 (41.2)		27,427 (44)	
	5-8	68,713 (30.7)		49,582 (30.7)		19,131 (30.7)	
	9+	61,217 (27.4)		45,390 (28.1)		15,827 (25.4)	
Number of chronic medical conditions, mean (SD)			7.9 (3.5)		8.0 (3.5)		7.6 (3.4)
**Number of chronic medical conditions, n (%)**							
	0-3	17,837 (8)		12,472 (7.7)		5365 (8.6)	
	4-7	95,444 (42.6)		67,335 (41.7)		28,109 (45.1)	
	8-11	76,940 (34.4)		56,135 (34.8)		20,805 (33.3)	
	12+	33,588 (15)		25,482 (15.8)		8106 (13)	
**At least 1emergency visit in the year prior to analysis period, n (%)**							
	No	120,972 (54.1)		85,801 (53.2)		35,171 (56.4)	
	Yes	102,837 (45.9)		75,623 (46.8)		27,214 (43.6)	
**At least 1 mental health visit in the year prior to analysis period, n (%)**							
	No	151,467 (67.7)		108,520 (67.2)		42,947 (68.8)	
	Yes	72,342 (32.3)		52,904 (32.8)		19,438 (31.2)	

^a^Figures in parentheses represent standard deviation for continuous variables and percentages for categorical variables.

^b^Telehealth comprises phone and video care.

However, there were several groups of veterans with higher adjusted odds of using cardiology telehealth care: most notably, women (adjusted odds ratio [AOR] 1.08, 95% CI 1.05-1.1); Hispanic or Latino veterans (AOR 1.46, 95% CI 1.43-1.49); and those with medium or long drive time (AOR 1.11, 95% CI 1.10-1.12 and AOR 1.09, 95% CI 1.07-1.10, respectively). Adjusted odds of using cardiology telehealth care differed minimally by age (for age 65-74 years compared to the reference category of age 18-49 years, AOR 0.97, 95% CI 0.94-0.99; adjusted odds for the other age categories were not statistically different from 1). Adjusted odds of using telehealth were slightly lower for rural and highly rural veterans compared to those living in urban settings (AOR 0.92, 95% CI 0.91-0.93 and AOR 0.93, 95% CI 0.91-0.96, respectively). Among veterans of different race and ethnicity groups, adjusted odds were lower for those of American Indian or Alaska Native or Black or African American race compared to White veterans (AOR 0.93, 95% CI 0.89-0.98 and AOR 0.97, 95% CI 0.96-0.98, respectively) and higher for those of unknown (to VHA) race (AOR 1.08, 95% CI 1.05-1.10). As above, we did see a larger-magnitude increase for veterans identifying as Hispanic or Latino, and to a lesser extent, unknown ethnicity (AOR 1.12, 95% CI 1.09-1.15).

Compared to those enrolled in VHA without special considerations, patients with low income had a slightly lower AOR for using cardiology telehealth care (AOR 0.96, 95% CI 0.94-0.97), as did those with low or moderate levels of disability (AOR 0.94, 95% CI 0.93-0.96); there was no difference from the reference group for those with high levels of disability. We also saw no differences in AOR for telehealth use among patients with a history of housing instability compared to those without such a history. Having a higher number of chronic health conditions was associated with slightly lower odds of using cardiology telehealth care compared to our reference of having 0-3 chronic conditions (4-7 conditions, AOR 0.95, 95% CI 0.94-0.97; 8-11 conditions, AOR 0.93, 95% CI 0.91-0.94; 12 or more conditions, AOR 0.93, 95% CI 0.91-0.95).

Examining relative proportions of variation in a patient’s likelihood of cardiology telehealth care use (ie, the intraclass correlation coefficient), 40.5% (95% CI 35.8%-45.3%) of variation was found at the patient level, 30.8% (95% CI 25.8%-36.2%) at the clinician level, and 7.0% (95% CI 6.3%-7.7%) at the facility level. In total, 21.7% of variation remained unexplained.

## Discussion

### Principal Findings

This analysis examines predictors of cardiology telehealth use among active users of cardiology care in this nation-wide integrated health care system. We found that patient-level characteristics explained the largest share of the attributable variability in VHA cardiology telehealth use. Clinician-level characteristics explained a more modest share, while facility-level factors contributed little to the variability seen.

Our findings may reflect a high level of standardization in telehealth-related policies (eg, around reimbursement) across VHA facilities. These results suggest that policy solutions intended to improve access and equity in use of cardiology telehealth care should focus on the patient and clinician levels. Such policy levers could include device or technical support for both patients and clinicians to increase the uptake of telehealth. For example, VHA has implemented a Digital Divide consult and tablet distribution program [22] to offer video-enabled devices to veterans with access barriers. Other strategies might include additional support staff to “room” a patient virtually and enter vital signs or chief complaint information; travel reimbursement for in-person appointments; and differential reimbursement for different visit modalities for clinicians, among many others. It should be noted that the desirability of increasing equity of telehealth use depends on the extent to which similar outcomes result from in-person and telehealth care. If the 2 modes are unequal in quality for a given use case, greater use of telehealth in a given subpopulation could represent worse access to quality care. Ongoing research comparing quality of telehealth and in-person care will help to unravel this issue.

The highest-yield drivers may be different in other environments. For example, Tzeng et al [11] found that facility-level factors accounted for more than clinician-level factors in total variance in outpatient virtual clinic use across multiple specialties in Taiwan. Rodriguez et al [23] found contributions to variance in the opposite order from our study (38% attributable to practices, 26% to clinicians, and 9% to patients) in video use (rather than telehealth use more generally) in primary and specialty care practices in the Mass General Brigham system. This variability underscores that drivers of telehealth use are likely to depend heavily on context: the level of the facility (department, practice, or medical center), whether the analysis focuses only on video or on all telehealth, and which specialties are included.

An important caveat when considering patient- versus clinician-level characteristics or policies is that it is challenging to completely disentangle factors at the level of patient versus clinician, as some subset of “patient” contribution may actually be clinician response to a given patient-level characteristic (eg, preferentially seeing older patients in person rather than via telehealth). To the extent that identified clinician-level variability reflects true clinician-level variation in practice, this raises the question of what the optimal breakdown of modalities ought to be for a given clinician, and how much variability in that breakdown across individuals is reasonable. Given that clinicians have differing preferences for telehealth use [24], are all clinicians obliged to offer some telehealth? Or is it acceptable—for quality and patient satisfaction—for some “virtualists” to offer mostly or exclusively telehealth [25] and others, primarily in-person care? These questions require further attention in the postpandemic period.

The reimbursement landscape for telehealth modalities will continue to evolve and affect the type of care offered. Reimbursing clinical time differentially depending on how care is delivered influences clinicians’ preferences around telehealth—even in integrated health care systems [8], where the clinician’s income is not directly impacted. If the Centers of Medicare and Medicaid (CMS) and health insurers choose to reimburse differently for video, telephone, and in-person visits, the drivers of telehealth use will undoubtedly change.

With regard to the association between telehealth use and other patient-level characteristics, consistent with other studies of VHA cardiology [6], Hispanic or Latino veterans had higher adjusted odds of cardiology telehealth use compared to not Hispanic or Latino individuals, and rural-dwelling veterans had lower odds of telehealth use compared to urban-dwellers. Earlier in the pandemic, we found that men were more likely than women to use cardiology telehealth care, an effect reversed in this study (potentially because of evolving patterns of use over time). Previously, we also found no association between telehealth use and long drive time, whereas in this study, those with a longer drive time were more likely to use telehealth. As in prior work examining disparities in cardiology telehealth care in VHA and across other health care systems, there were small or no differences by race [6,7,26]; whether or not differences in telehealth use appear by age has varied across systems [6,7,26].

### Limitations

Our study has limitations common to observational data and work in VHA’s system. First, generalizability beyond VHA—a national, integrated health care system with long-standing, well-established processes in place to standardize practices across facilities—or to populations with less engaged users of care may be limited. Second, because we constructed our facility-level variable at the VHA medical center level, we cannot assess whether more granular facility levels (eg, individual clinics) could explain additional variation in telehealth use. Third, while all patients included had at least 1 cardiology diagnosis, we lack the specific diagnoses that were being addressed at these encounters. Fourth, we have analyzed telehealth visits with phone and video visits combined; in this population, video visits were rare enough that 3-level multilevel models were not feasible, and thus we cannot specifically report on the differential contributions to variability in video care use or whether those differ from the pattern among all telehealth visits. Fifth, our results represent a pooled estimate across the study period of 2020-2022, while use patterns may have varied across the pandemic [2,27]. Sixth, we do not include facility-level or provider-level characteristics due to computational limitations and data availability. Finally, we attributed patients to a single clinician and site to allow our statistical models to run; as a majority of veterans did see more than 1 cardiology provider during the study period, this required a significant simplification of our complex real-world data.

### Conclusions

In sum, within VHA, a nationally integrated health care system, there are marked differences in the degree to which patient, clinician, and facility factors influence use of cardiology telehealth care. Given that most variability occurs at the patient and clinician levels, it suggests that these levels might be optimal targets for interventions intended to alter the mix of telehealth versus in-person care use.

## Appendix

Multimedia Appendix 1 Cardiac Diagnosis Codes; Stop Codes; Chronic Condition Groups; Full Results of Multilevel Multivariable Logistic Regression Model; Variability in Utilization and Volume across Patients, Providers, and Facilities.

## Abbreviations

CMS: Centers for Medicare and Medicaid Services
ICD-10: International Classification of Disease, Tenth Revision
NPI: National Provider Identifier
PSSG: Planning Systems Support Group
VHA: Veterans Health Administration

## Data Availability

Due to US Department of Veterans Affairs (VA) regulations and our ethics agreements, the analytic datasets used for this study are not permitted to leave the VA firewall without a Data Use Agreement. This limitation is consistent with other studies based on VA data. However, VA data are made freely available to researchers with an approved VA study protocol.
