# Supplementary material for: Patient-, Provider-, and Facility-Level Contributors to the Use of Cardiology Telehealth Care in the Veterans Health Administration: Retrospective Cohort Study
Source: J Med Internet Res. 2024 Oct 25;26:e53298. doi: 10.2196/53298 (PMC11549580; doi:10.2196/53298)
Supplement: Multimedia Appendix 1 [file jmir_v26i1e53298_app1.docx]

**Table S1. ICD-10 Codes for Included Cardiac Diagnoses**

| **Condition** | **ICD-10 Codes** |
| --- | --- |
| Arrhythmia/  Conduction Disorder | G90.01,I44.0,I44.1,I44.2,I44.30,I44.39,I44.4,I44.5,I44.60,I44.69,I44.7,I45.0,I45.10,I45.19,I45.2,I45.3,I45.4,I45.5,I45.6,I45.81,I45.89,I45.9,I46.2,I46.8,I46.9,I47.0,I47.1,I47.2,I47.9,I48.0,I48.1,I48.2,I48.3,I48.4,I48.91,I48.92,I49.01,I49.02,I49.1,I49.2,I49.3,I49.40,I49.49,I49.5,I49.8,I49.9,Q24.6,R00.0,R00.1,R00.2,T82.110A,T82.111A,T82.120A,T82.121A,T82.190A,T82.191A,Z45.010,Z45.018,Z45.02,Z86.74,Z95.0,Z95.810,Z95.9 |
| Cerebrovascular Disease | G43.601,G43.609,G43.611,G43.619,G45.0,G45.1,G45.2,G45.8,G45.9,G46.0,G46.1,G46.2,G46.3,G46.4,G46.5,G46.6,G46.7,G46.8,I63.00,I63.011,I63.012,I63.013,I63.019,I63.02,I63.031,I63.032,I63.033,I63.039,I63.09,I63.10,I63.111,I63.112,I63.113,I63.119,I63.12,I63.131,I63.132,I63.133,I63.139,I63.19,I63.20,I63.211,I63.212,I63.213,I63.219,I63.22,I63.231,I63.232,I63.233,I63.239,I63.29,I63.30,I63.311,I63.312,I63.313,I63.319,I63.321,I63.322,I63.323,I63.329,I63.331,I63.332,I63.333,I63.339,I63.341,I63.342,I63.343,I63.349,I63.39,I63.40,I63.411,I63.412,I63.413,I63.419,I63.421,I63.422,I63.423,I63.429,I63.431,I63.432,I63.433,I63.439,I63.441,I63.442,I63.443,I63.449,I63.49,I63.50,I63.511,I63.512,I63.513,I63.519,I63.521,I63.522,I63.523,I63.529,I63.531,I63.532,I63.533,I63.539,I63.541,I63.542,I63.543,I63.549,I63.59,I63.6,I63.8,I63.9,I65.01,I65.02,I65.03,I65.09,I65.1,I65.21,I65.22,I65.23,I65.29,I65.8,I65.9,I66.01,I66.02,I66.03,I66.09,I66.11,I66.12,I66.13,I66.19,I66.21,I66.22,I66.23,I66.29,I66.3,I66.8,I66.9,I67.1,I67.2,I67.5,I67.6,I67.7,I67.81,I67.82,I67.841,I67.848,I67.89,I67.9,I68.0,I68.2,I68.8,I69.00,I69.01,I69.010,I69.011,I69.012,I69.013,I69.014,I69.015,I69.018,I69.019,I69.020,I69.021,I69.022,I69.023,I69.028,I69.031,I69.032,I69.033,I69.034,I69.039,I69.041,I69.042,I69.043,I69.044,I69.049,I69.051,I69.052,I69.053,I69.054,I69.059,I69.061,I69.062,I69.063,I69.064,I69.065,I69.069,I69.090,I69.091,I69.092,I69.093,I69.098,I69.10,I69.11,I69.110,I69.111,I69.112,I69.113,I69.114,I69.115,I69.118,I69.119,I69.120,I69.121,I69.122,I69.123,I69.128,I69.131,I69.132,I69.133,I69.134,I69.139,I69.141,I69.142,I69.143,I69.144,I69.149,I69.151,I69.152,I69.153,I69.154,I69.159,I69.161,I69.162,I69.163,I69.164,I69.165,I69.169,I69.190,I69.191,I69.192,I69.193,I69.198,I69.20,I69.21,I69.210,I69.211,I69.212,I69.213,I69.214,I69.215,I69.218,I69.219,I69.220,I69.221,I69.222,I69.223,I69.228,I69.231,I69.232,I69.233,I69.234,I69.239,I69.241,I69.242,I69.243,I69.244,I69.249,I69.251,I69.252,I69.253,I69.254,I69.259,I69.261,I69.262,I69.263,I69.264,I69.265,I69.269,I69.290,I69.291,I69.292,I69.293,I69.298,I69.30,I69.31,I69.310,I69.311,I69.312,I69.313,I69.314,I69.315,I69.318,I69.319,I69.320,I69.321,I69.322,I69.323,I69.328,I69.331,I69.332,I69.333,I69.334,I69.339,I69.341,I69.342,I69.343,I69.344,I69.349,I69.351,I69.352,I69.353,I69.354,I69.359,I69.361,I69.362,I69.363,I69.364,I69.365,I69.369,I69.390,I69.391,I69.392,I69.393,I69.398,I69.80,I69.81,I69.810,I69.811,I69.812,I69.813,I69.814,I69.815,I69.818,I69.819,I69.820,I69.821,I69.822,I69.823,I69.828,I69.831,I69.832,I69.833,I69.834,I69.839,I69.841,I69.842,I69.843,I69.844,I69.849,I69.851,I69.852,I69.853,I69.854,I69.859,I69.861,I69.862,I69.863,I69.864,I69.865,I69.869,I69.890,I69.891,I69.892,I69.893,I69.898,I69.90,I69.91,I69.910,I69.911,I69.912,I69.913,I69.914,I69.915,I69.918,I69.919,I69.920,I69.921,I69.922,I69.923,I69.928,I69.931,I69.932,I69.933,I69.934,I69.939,I69.941,I69.942,I69.943,I69.944,I69.949,I69.951,I69.952,I69.953,I69.954,I69.959,I69.961,I69.962,I69.963,I69.964,I69.965,I69.969,I69.990,I69.991,I69.992,I69.993,I69.998,I97.810,I97.811,I97.820,I97.821,Q28.2,Q28.3,Z86.73 |
| Coronary Artery Disease | I20.0,I21.01,I21.02,I21.09,I21.11,I21.19,I21.21,I21.29,I21.3,I21.4,I21.9,I21.A1,I21.A9,I22.0,I22.1,I22.2,I22.8,I22.9,I23.0,I23.1,I23.2,I23.3,I23.6,I23.7,I23.8,I24.0,I24.1,I24.8,I24.9,I25.10,I25.110,I25.111,I25.118,I25.119,I25.2,I25.3,I25.41,I25.42,I25.5,I25.6,I25.700,I25.710,I25.720,I25.730,I25.750,I25.760,I25.790,I25.810,I25.811,I25.812,I25.82,I25.83,I25.84,I25.89,I25.9,I51.0,T82.211A,T82.212A,T82.213A,T82.218A,Z95.1,Z95.5,Z98.61 |
| Heart Failure | I09.81,I27.0,I27.1,I27.2,I27.20,I27.21,I27.22,I27.23,I27.24,I27.29,I27.81,I27.89,I27.9,I42.0,I42.1,I42.2,I42.3,I42.4,I42.5,I42.6,I42.7,I42.8,I42.9,I43.,I50.1,I50.20,I50.21,I50.22,I50.23,I50.30,I50.31,I50.32,I50.33,I50.40,I50.41,I50.42,I50.43,I50.810,I50.811,I50.812,I50.813,I50.814,I50.82,I50.83,I50.84,I50.89,I50.9,I51.5,I51.7,I51.81,Z48.21,Z48.280,Z94.1,Z94.3,Z95.812 |
| Hypertension | H35.031,H35.032,H35.033,H35.039,I10.,I11.0,I11.9,I12.0,I12.9,I13.0,I13.10,I13.11,I13.2,I15.0,I15.1,I15.2,I15.8,I15.9,I16.0,I16.1,I16.9,I67.4,N26.2 |
| Lipid Disorders | E78.0,E78.00,E78.01,E78.1,E78.2,E78.3,E78.4,E78.5 |
| Valvular Disease | A39.51,B33.21,B37.6,I01.1,I05.0,I05.1,I05.2,I05.8,I05.9,I06.0,I06.1,I06.2,I06.8,I06.9,I07.0,I07.1,I07.2,I07.8,I07.9,I08.0,I08.1,I08.2,I08.3,I08.8,I08.9,I09.1,I23.4,I23.5,I33.0,I33.9,I34.0,I34.1,I34.2,I34.8,I34.9,I35.0,I35.1,I35.2,I35.8,I35.9,I36.0,I36.1,I36.2,I36.8,I36.9,I37.0,I37.1,I37.2,I37.8,I37.9,I38.,I39.,I51.1,I51.2,Q22.0,Q22.1,Q22.2,Q22.3,Q23.0,Q23.1,Q23.2,Q23.3,Q24.3,R01.0,R01.1,T82.01XA,T82.02XA,T82.03XA,T82.09XA,Z95.2,Z95.3,Z95.4 |
| Vascular Disease | D35.5,E08.51,E08.52,E09.51,E09.52,E10.51,E10.52,I26.01,I26.90,I28.0,I28.1,I28.8,I28.9,I67.0,I70.0,I70.1,I70.201,I70.202,I70.203,I70.208,I70.209,I70.211,I70.212,I70.213,I70.218,I70.219,I70.221,I70.222,I70.223,I70.228,I70.229,I70.231,I70.241,I70.261,I70.262,I70.263,I70.268,I70.269,I70.291,I70.292,I70.293,I70.298,I70.299,I70.301,I70.302,I70.303,I70.308,I70.309,I70.311,I70.312,I70.313,I70.318,I70.319,I70.321,I70.322,I70.323,I70.328,I70.329,I70.361,I70.362,I70.363,I70.368,I70.369,I70.391,I70.392,I70.393,I70.398,I70.399,I70.401,I70.402,I70.403,I70.408,I70.409,I70.411,I70.412,I70.413,I70.418,I70.419,I70.421,I70.422,I70.423,I70.428,I70.429,I70.461,I70.462,I70.463,I70.468,I70.469,I70.491,I70.492,I70.493,I70.498,I70.499,I70.501,I70.502,I70.503,I70.508,I70.509,I70.511,I70.512,I70.513,I70.518,I70.519,I70.521,I70.522,I70.523,I70.528,I70.529,I70.561,I70.562,I70.563,I70.568,I70.569,I70.591,I70.592,I70.593,I70.598,I70.599,I70.601,I70.602,I70.603,I70.608,I70.609,I70.611,I70.612,I70.613,I70.618,I70.619,I70.621,I70.622,I70.623,I70.628,I70.629,I70.661,I70.662,I70.663,I70.668,I70.669,I70.691,I70.692,I70.693,I70.698,I70.699,I70.701,I70.702,I70.703,I70.708,I70.709,I70.711,I70.712,I70.713,I70.718,I70.719,I70.721,I70.722,I70.723,I70.728,I70.729,I70.761,I70.762,I70.763,I70.768,I70.769,I70.791,I70.792,I70.793,I70.798,I70.799,I70.8,I70.90,I70.91,I70.92,I71.00,I71.01,I71.02,I71.03,I71.1,I71.2,I71.3,I71.4,I71.5,I71.6,I71.8,I71.9,I72.0,I72.1,I72.2,I72.3,I72.4,I72.5,I72.6,I72.8,I72.9,I73.1,I73.89,I73.9,I74.01,I74.09,I74.10,I74.11,I74.19,I74.2,I74.3,I74.4,I74.5,I74.8,I74.9,I75.011,I75.012,I75.013,I75.019,I75.021,I75.022,I75.023,I75.029,I75.81,I75.89,I77.0,I77.1,I77.2,I77.3,I77.4,I77.5,I77.6,I77.70,I77.71,I77.72,I77.73,I77.74,I77.75,I77.76,I77.77,I77.79,I77.810,I77.811,I77.812,I77.819,I77.89,I77.9,I78.0,I78.8,I78.9,I79.0,I79.1,I79.8,I80.00,I80.01,I80.02,I80.03,I80.10,I80.11,I80.12,I80.13,I80.201,I80.202,I80.203,I80.209,I80.211,I80.212,I80.213,I80.219,I80.221,I80.222,I80.223,I80.229,I80.231,I80.232,I80.233,I80.239,I80.291,I80.292,I80.293,I80.299,I80.3,I80.8,I80.9,I81.,I82.0,I82.1,I82.601,I82.602,I82.603,I82.609,I82.611,I82.612,I82.613,I82.619,I82.701,I82.702,I82.703,I82.709,I82.711,I82.712,I82.713,I82.719,I82.811,I82.812,I82.813,I82.819,I82.890,I82.891,I82.90,I82.91,I83.001,I83.002,I83.003,I83.004,I83.005,I83.008,I83.009,I83.011,I83.012,I83.013,I83.014,I83.015,I83.018,I83.019,I83.021,I83.022,I83.023,I83.024,I83.025,I83.028,I83.029,I83.10,I83.11,I83.12,I83.201,I83.202,I83.203,I83.204,I83.205,I83.208,I83.209,I83.211,I83.212,I83.213,I83.214,I83.215,I83.218,I83.219,I83.221,I83.222,I83.223,I83.224,I83.225,I83.228,I83.229,I83.811,I83.812,I83.813,I83.819,I83.891,I83.892,I83.893,I83.899,I83.90,I83.91,I83.92,I83.93,I86.0,I86.1,I86.2,I86.3,I86.4,I86.8,I87.001,I87.002,I87.003,I87.009,I87.011,I87.012,I87.013,I87.019,I87.021,I87.022,I87.023,I87.029,I87.031,I87.032,I87.033,I87.039,I87.091,I87.092,I87.093,I87.099,I87.1,I87.2,I87.301,I87.302,I87.303,I87.309,I87.311,I87.312,I87.313,I87.319,I87.321,I87.322,I87.323,I87.329,I87.331,I87.332,I87.333,I87.339,I87.391,I87.392,I87.393,I87.399,I96.,K55.0,K55.011,K55.012,K55.019,K55.021,K55.022,K55.029,K55.031,K55.032,K55.039,K55.041,K55.042,K55.049,K55.051,K55.052,K55.059,K55.061,K55.062,K55.069,K55.1,K55.30,K55.31,K55.32,K55.33,K55.8,K55.9,M31.8,M31.9,P29.3,P29.30,P29.38,Q25.1,Q25.2,Q25.21,Q25.29,Q25.3,Q25.4,Q25.40,Q25.41,Q25.42,Q25.43,Q25.44,Q25.45,Q25.46,Q25.47,Q25.48,Q25.49,Q25.5,Q25.6,Q25.71,Q25.72,Q25.79,Q25.8,Q25.9,Q26.0,Q26.1,Q26.2,Q26.3,Q26.4,Q26.5,Q26.6,Q26.8,Q26.9,Q27.0,Q27.1,Q27.2,Q27.31,Q27.32,Q27.33,Q27.34,Q27.39,Q27.8,Q27.9,T82.310A,T82.311A,T82.312A,T82.318A,T82.319A,T82.320A,T82.321A,T82.322A,T82.328A,T82.329A,T82.330A,T82.331A,T82.332A,T82.338A,T82.339A,T82.390A,T82.391A,T82.392A,T82.398A,T82.399A,T82.41XA,T82.42XA,T82.43XA,T82.49XA,T82.510A,T82.511A,T82.513A,T82.514A,T82.515A,T82.518A,T82.520A,T82.521A,T82.523A,T82.524A,T82.525A,T82.528A,T82.529A,T82.530A,T82.531A,T82.533A,T82.534A,T82.535A,T82.538A,T82.590A,T82.591A,T82.593A,T82.594A,T82.595A,T82.598A,T82.7XXA,T82.818A,T82.828A,T82.838A,T82.848A,T82.856A,T82.858A,T82.868A,T82.898A,T82.9XXA,Z86.72,Z95.820,Z95.828 |

**Table S2. Stop codes for categorization of encounter types**

| **Category** | **Stop Code(s) and/or ICD-10 Codes** |
| --- | --- |
| Cardiology Visit | 303 in primary position OR in secondary position with telephone visit stop code in primary position |
| Video | 179: Real Time Clinical Video Telehealth To Home - Provider Site, 648: Real Time Clinical Video Telehealth With Non-VAMC Location - Provider Site, 679: National Center Real Time Clinical Video Telehealth To Home- Provider Site |
| Phone | 324 Telephone/Medicine |
| Emergency Department/ Urgent Care | 130,131,297 |
| Mental Health | 156,157,292,502,503,504,505,506,507,508,509,510,511,512,513,514,516,519,522,523,524,525,527,528,529,530,531,533,534,535,536,538,539,540,542,545,546,547,548,550,552,553,554,555,556,557,558,560,561,562,564,565,566,567,568,571,572,573,574,575,576,577,579,580,582,583,584,586,587,588,590,591,592,593,595,596,597,598,599,707,713 |
| Other | 103,104,105,106,107,108,109,110,111,115,116,117,123,124,125,126,127,128,135,136,137,139,142,144,145,146,147,148,149,150,151,153,154,155,158,159,160,162,165,166,167,168,169,179,180,181,182,183,184,185,186,187,188,189,192,212,320,328,332,370,371,372,373,421,436,440,443,444,445,446,447,448,449,450,457,474,481,490,491,499,644,645,646,647,648,649,651,652,653,656,669,673,674,679,683,684,685,686,690,692,693,694,695,696,697,698,699,701,703,706,708,710,714,716,717,719,720,721,722,723,724,801,802,803,901,999 |
| Primary Care | 170,171,172,301,318,322,323,338,341,342,348,350,704 |
| Rehabilitation Services | 195,196,197,198,199,201,202,203,204,205,206,207,208,209,210,211,213,214,215,216,217,218,220,221,222,224,225,229,230,240,241,250,295,296,417,423,425,437,438,439 |
| Specialty Care | 102,118,119,120,121,143,173,174,175,176,177,178,190,191,231,290,291,293,302,303,304,305,306,307,308,309,310,311,312,313,314,315,316,317,319,321,324,325,326,327,329,330,331,333,334,335,336,337,339,340,344,345,346,347,349,351,352,353,354,356,369,391,392,394,401,402,403,404,405,406,407,408,409,410,411,412,413,414,415,416,418,419,420,422,424,426,427,428,429,430,431,432,433,434,435,441,486,487,488,489,602,603,604,606,607,608,610,611,658,680,681,682,718 |
| Housing Instability/ History of Homelessness* | 504,507,508,511,522,528,529,530,555,556,590, Z59.0,Z59.1,Z59.9 |

*Housing instability/history of homelessness includes both stop codes and ICD-10 codes.

**Table S3. Clinical Condition Groups (based on Yoon et al 2018 (1))**

| **Condition** |
| --- |
| Allergic and Other Chronic Sinusitis/Rhinitis |
| Anemia |
| Arrhythmia/Conduction Disorder |
| Asthma |
| Cancer - all types |
| Cerebrovascular Disease |
| Chronic Obstructive Pulmonary Disease |
| Chronic Pain Syndromes |
| Coagulation and Hemorrhagic Disorders |
| Coronary Artery Disease |
| Dementia |
| Diabetes Mellitus |
| Epilepsy/Convulsions |
| Esophageal/Gastric/Duodenal Disorders |
| Heart Failure |
| HIV/AIDS |
| Hypertension |
| Inflammatory Bowel Disease |
| Joint Disorders |
| Lipid Disorders |
| Liver Disease or Hepatitis C |
| Multiple Sclerosis |
| Osteoporosis |
| Overweight/Obesity |
| Parkinson's Disease |
| Peripheral Nerve Disorders |
| Renal Failure or Nephropathy |
| Rheumatologic/Autoimmune Disorders |
| Spinal Cord Injury or Paralysis |
| Spine Disorders |
| Thrombocytopenia |
| Thyroid disorders |
| Tobacco Use Disorder |
| Traumatic Brain Injury |
| Tuberculosis |
| Valvular Disease |
| Vascular Disease |
| Alcohol Use Disorders |
| Anxiety Disorders - Other |
| Bipolar Disorders |
| Depression |
| Drug Use Disorders, non-opioid |
| Drug Use Disorders, opioid |
| Personality Disorders |
| Psychotic Disorders - Other |
| PTSD |
| Schizophrenia |

**Table S4. Full Results of Multilevel Multivariable Logistic Regression Model**

|  | **Adjusted Odds Ratio** | **95% Confidence Interval** |
| --- | --- | --- |
| **Age, years, categorical** |  | |
| **18-49** | Reference | |
| **50-64** | 0.99 | [0.96,1.01] |
| **65-74** | 0.97 | [0.94,0.99] |
| **75+** | 1.00 | [0.97,1.03] |
| **Race** |  | |
| **American Indian or Alaska Native** | 0.93 | [0.89,0.98] |
| **Asian** | 1.05 | [0.99,1.11] |
| **Black or African American** | 0.97 | [0.96,0.98] |
| **Native Hawaiian or other Pacific Islander** | 1.00 | [0.96,1.05] |
| **Unknown** | 1.08 | [1.05,1.10] |
| **White** | Reference | |
| **Ethnicity** |  | |
| **Not Hispanic or Latino** | Reference | |
| **Hispanic or Latino** | 1.46 | [1.43,1.49] |
| **Unknown** | 1.12 | [1.09,1.15] |
| **Gender** |  | |
| **Female** | 1.08 | [1.05,1.10] |
| **Male** | Reference | |
| **Rurality** |  | |
| **Urban** | Reference | |
| **Rural** | 0.92 | [0.91,0.93] |
| **Highly Rural** | 0.93 | [0.91,0.96] |
| **Drive Time to Secondary Care, categorical** |  | |
| **Short** | Reference | |
| **Medium** | 1.11 | [1.10,1.12] |
| **Long** | 1.09 | [1.07,1.10] |
| **Missing** | 1.05 | [1.00,1.11] |
| **Enrollment Priority** |  | |
| **No service disability** | Reference | |
| **Low/moderate disability** | 0.96 | [0.94,0.97] |
| **High disability** | 0.94 | [0.93,0.96] |
| **Low income** | 0.99 | [0.98,1.01] |
| **History of Housing Instability/Homelessness** |  | |
| **No history of housing instability** | Reference | |
| **History of housing instability** | 1.01 | [0.99,1.04] |
| **Primary care visits in year prior to analysis period, categorical** |  | |
| **0-4** | Reference | |
| **5-8** | 1.02 | [1.00,1.03] |
| **9+** | 1.04 | [1.02,1.05] |
| **Number of chronic medical conditions, categorical** |  | |
| **0-3** | Reference | |
| **4-7** | 0.95 | [0.94,0.97] |
| **8-11** | 0.93 | [0.91,0.94] |
| **12+** | 0.93 | [0.91,0.95] |
| **At least one emergency visit in the year prior to analysis period** |  | |
| **No** | Reference | |
| **Yes** | 0.98 | [0.97,0.99] |
| **At least one mental health visit in the year prior to analysis period** |  | |
| **No** | Reference | |
| **Yes** | 1.02 | [1.01,1.03] |
| **Calendar Year** |  | |
| **2020** | Reference | |
| **2021** | 0.31 | [0.31,0.31] |
| **2022** | 0.23 | [0.22,0.23] |
|  | | |
| **Residual Intraclass Correlation** |  |  |
| **VA Medical Center** | .07 | [0.06,0.08] |
| **Main Cardiology Provider \| VA Medical Center** | 0.31 | [0.26,0.36] |
| **Patient \| Main Cardiology Provider \| VA Medical Center** | 0.40 | [0.36,0.45] |

Exponentiated coefficients; 95% confidence intervals in brackets

**Figure S1. Variability in Utilization and Volume across Patients, Providers, and Facilities^a^**


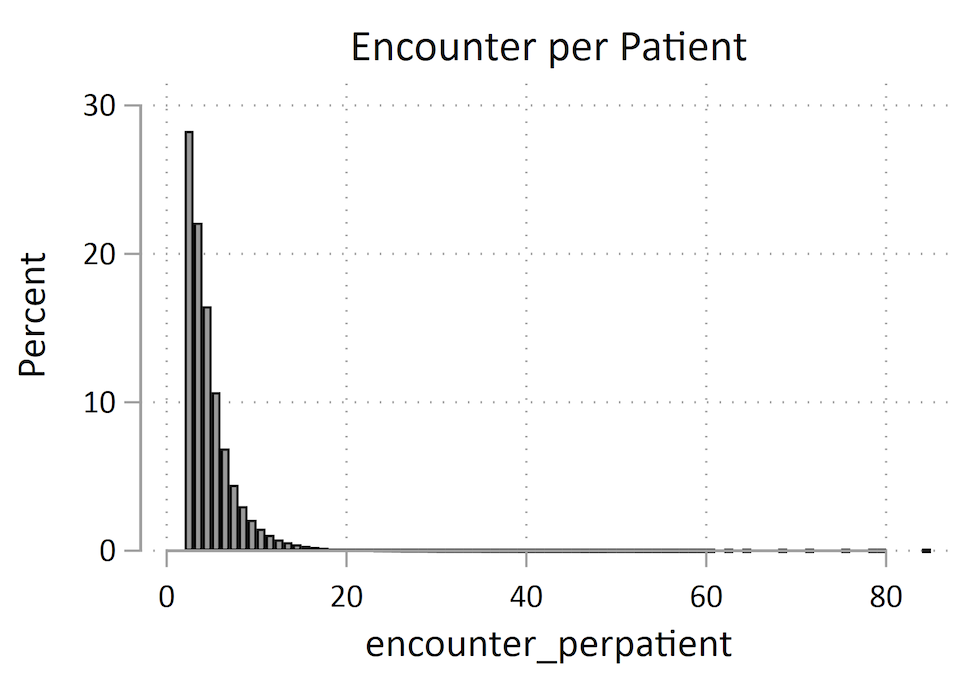

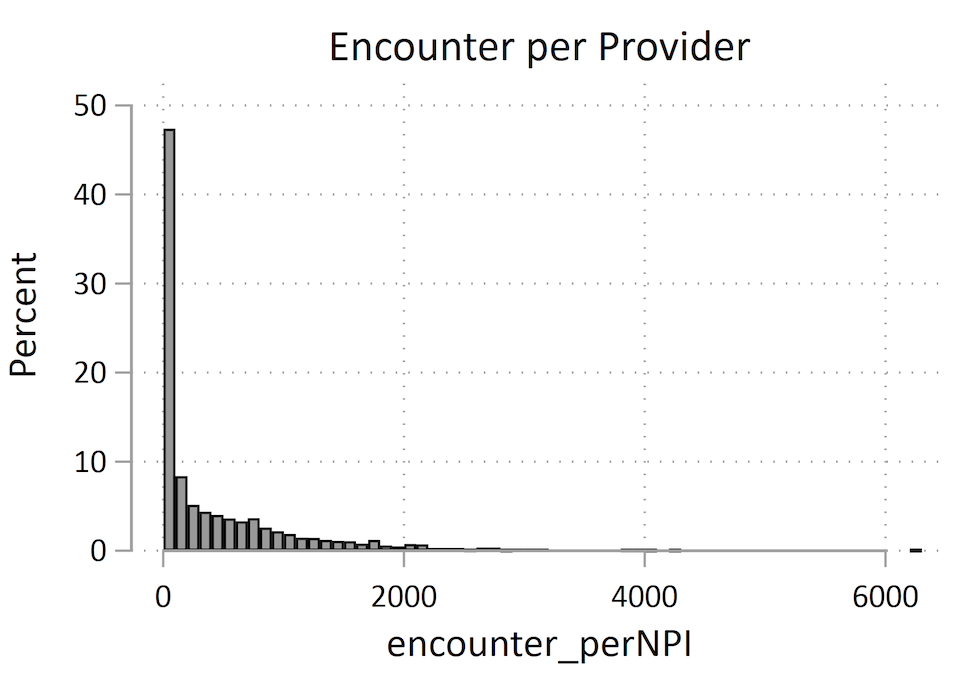


Cardiology Encounters per Patient Cardiology Encounters per Provider


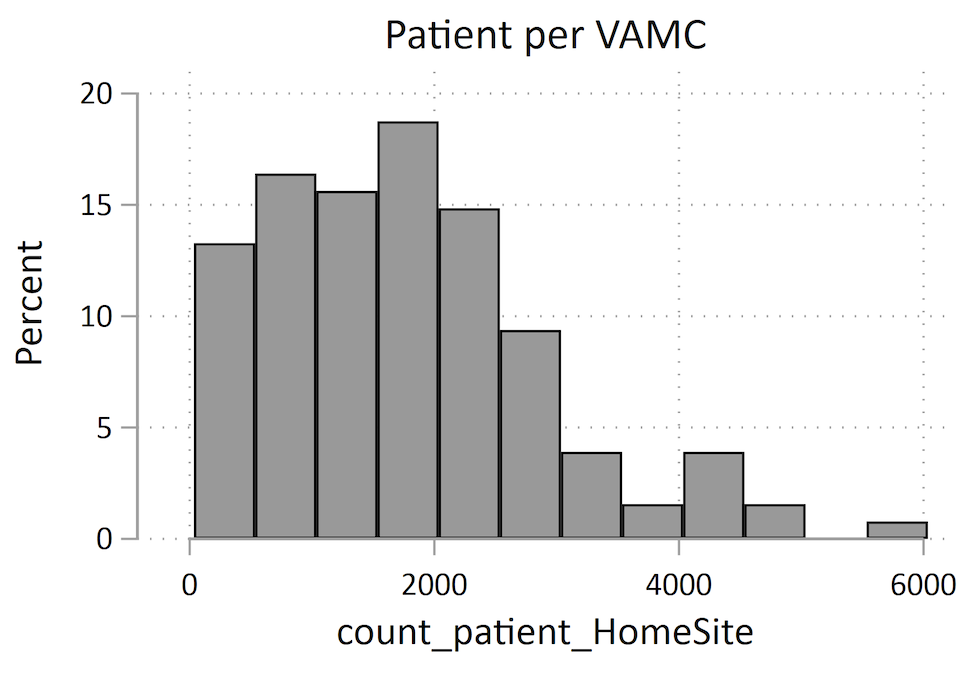

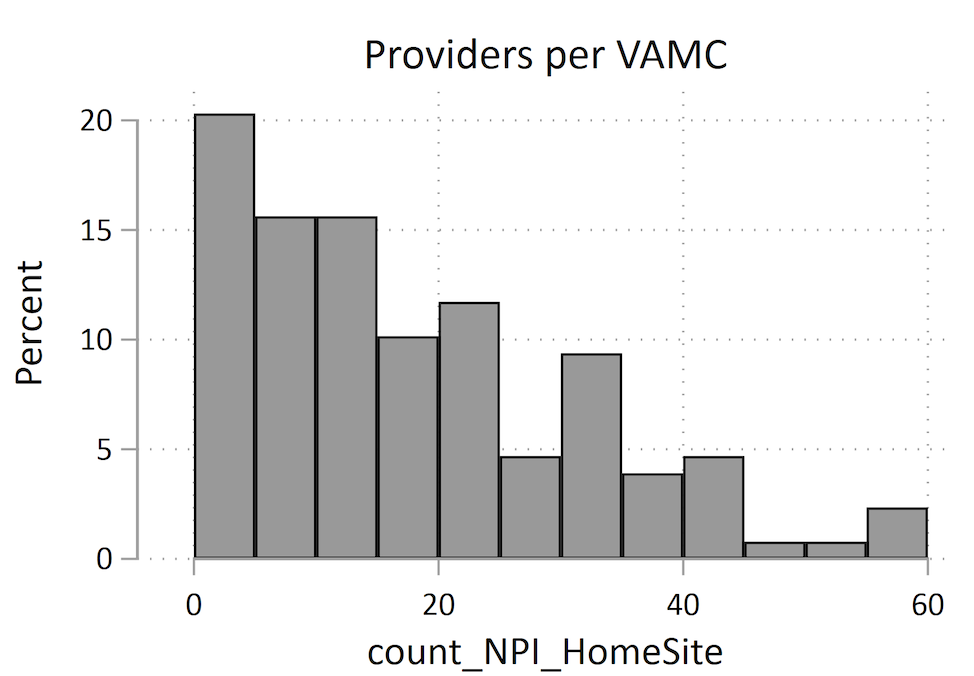


Cardiology Patients per VA Medical Center Cardiology Providers per VA Medical Center

^a^ Note that these patients are limited to those included in the study cohort, i.e., engaged in cardiology care prior to the pandemic, and reflect those patients’ encounters and providers only.

**Supplemental References**

1. Yoon J, Chee CP, Su P, Almenoff P, Zulman DM, Wagner TH. Persistence of High Health Care Costs among VA Patients. Health Serv Res. 2018 Oct;53(5):3898–916.
